# PSMA PET/CT–Derived Indicators and Outcomes After [^177^Lu]Lu-PSMA-617: A Multicenter Retrospective Analysis from the U.S. Expanded-Access Program

**DOI:** 10.2967/jnumed.125.270789

**Published:** 2026-05

**Authors:** Koichiro Kimura, Vishnu Murthy, Andrew F. Voter, Lilja B. Solnes, Abuzar Moradi Tuchayi, Surekha Yadav, Lela Theus, Andrew Nguyen, Vinicius Ludwig, Adrien Holzgreve, Lena M. Unterrainer, Tristan R. Grogan, Johannes Czernin, Thomas A. Hope, Andrei Gafita, Jeremie Calais

**Affiliations:** 1 Department of Nuclear Medicine and Theranostics, Ahmanson Translational Theranostics Division, David Geffen School of Medicine at UCLA, Los Angeles, California;; 2 Division of Nuclear Medicine and Molecular Imaging, The Russell H. Morgan Department of Radiology and Radiological Science, Johns Hopkins University School of Medicine, Baltimore, Maryland;; 3 Department of Radiology and Biomedical Imaging, University of California, San Francisco, California;; 4 Department of Nuclear Medicine, LMU University Hospital, LMU Munich, Munich, Germany;; 5 Department of Nuclear Medicine, Technical University of Munich, Munich, Germany; and; 6 Department of Medicine Statistics Core, David Geffen School of Medicine at UCLA, Los Angeles, California

**Keywords:** metastatic castration-resistant prostate cancer, PSMA PET/CT, ^177^Lu-PSMA, imaging biomarker, SUV_mean_

## Abstract

Pretherapeutic visual and quantitative indicators derived from prostate-specific membrane antigen (PSMA) PET/CT have been proposed as predictors of response to [^177^Lu]Lu-PSMA-617 (^177^Lu-PSMA) therapy in patients with metastatic castration-resistant prostate cancer. This study aimed to evaluate and compare the prognostic performance of these indicators in a cohort treated under the U.S. Expanded-Access Program. **Methods:** This retrospective analysis included 88 patients with metastatic castration-resistant prostate cancer from 3 U.S. institutions (University of California Los Angeles, University of California San Francisco, and Johns Hopkins) enrolled in the Expanded-Access Program who underwent baseline PSMA PET/CT before receiving ^177^Lu-PSMA. We assessed visual indicators—such as the visual PSMA PET tumor–to–salivary gland ratio, tumor heterogeneity, and intensity scores—and quantitative metrics including total tumor volume (TTV), total tumor SUV_mean_, total tumor SUV_max_, total lesion uptake (TTV × total tumor SUV_mean_), total lesion quotient (TTV ÷ total tumor SUV_mean_), and the quantitative PSMA PET tumor–to–salivary gland score. Associations with clinical outcomes—a 50% or greater prostate-specific antigen decline (PSA50), prostate-specific antigen (PSA) progression-free survival (PFS), and overall survival (OS)—were analyzed using univariate and multivariate models. Predictive performance was evaluated via the concordance index from Cox proportional hazards regression. **Results:** After a median follow-up of 36.1 mo (95% CI, 33.8–37.8 mo), the PSA50 rate was 43%, the median PSA PFS was 4.5 mo (95% CI, 3.7–7.2 mo), and the 2-y PSA PFS rate was 7% (95% CI, 3–16%). The median OS was 12.5 mo (95% CI, 10.4–17.1 mo), and the 2-y OS rate was 29% (95% CI, 20–40%). Among the evaluated metrics, total tumor SUV_mean_ showed the highest predictive accuracy for PSA50 (area under the curve, 0.81; 95% CI, 0.73–0.90). In multivariate analyses adjusted for clinical factors, a higher total tumor SUV_mean_ was independently associated with improved PSA PFS (hazard ratio, 0.58; 95% CI, 0.39–0.86; *P* = 0.007) and OS (hazard ratio, 0.54; 95% CI, 0.34–0.86; *P* = 0.009). Total tumor SUV_mean_ also yielded higher concordance index values compared with models based on clinical variables alone (PSA PFS, 0.667 vs. 0.594; OS, 0.687 vs. 0.661, respectively). **Conclusion:** Baseline total tumor SUV_mean_ on PSMA PET/CT provides independent prognostic information beyond clinical parameters and may serve as a useful biomarker for patient selection and treatment personalization with ^177^Lu-PSMA.

## Visual Abstract

**Figure:**
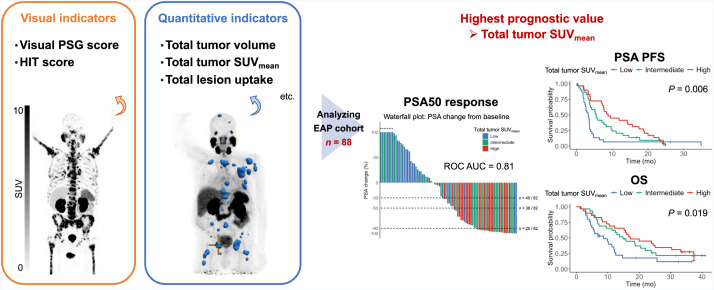

Therapy with [^177^Lu]Lu-PSMA-617 (^177^Lu-PSMA) has been shown to prolong progression-free survival (PFS) and overall survival (OS) of patients with end-stage metastatic castration-resistant prostate cancer (mCRPC) (1). However, the efficacy of ^177^Lu-PSMA is not uniform; thus, various imaging-based biomarkers derived from pretherapeutic baseline prostate-specific membrane antigen (PSMA) PET/CT have been proposed to better identify patients who will likely benefit from ^177^Lu-PSMA (2,3). International guidelines for ^177^Lu-PSMA emphasize the impact of pretherapeutic PSMA PET/CT for appropriate patient selection (4). Multiple studies, which include secondary analysis of the VISION and TheraP trials, demonstrated that the total tumor SUV_mean_ on pretherapeutic baseline PSMA PET, as a quantitative indicator, is associated with better outcomes after treatment with ^177^Lu-PSMA (5–10). In addition, studies suggested visual indicators as useful surrogate markers for SUV_mean_ that can be easily implemented into clinical practice as they mainly rely on the maximum-intensity-projection images from the PSMA PET and can be performed without a dedicated segmentation software (11,12). Yet, imaging-derived biomarkers have not yet been widely incorporated into clinical decision-making for ^177^Lu-PSMA therapy, primarily because of limited evidence supporting their reproducibility and incremental prognostic value beyond established clinical parameters such as serum prostate-specific antigen (PSA) levels, other laboratory markers, and patient characteristics.

The U.S. Expanded Access Program (EAP; NCT04825652), initiated in April 2021 to provide ^177^Lu-PSMA to eligible patients with mCRPC before Food and Drug Administration regulatory approval, enrolled a broader and more diverse patient population than typical clinical trials, because of the greater flexibility in patient enrollment, while still maintaining eligibility criteria aligned with the VISION trial. The aim of the present study was to test various proposed baseline PSMA PET/CT–derived outcome indicators within the U.S. EAP cohort and confirm whether these imaging biomarkers add value to clinical information at the pretreatment stage of ^177^Lu-PSMA.

## MATERIALS AND METHODS

### Patients

This retrospective analysis included 88 patients with mCRPC enrolled in the EAP at 3 institutions across the U.S. (University of California Los Angeles [61/88, 69%], University of California San Francisco [9/88, 10%], and Johns Hopkins [18/88, 21%]) with pretherapeutic [^68^Ga]Ga-PSMA-11 or [^18^F]F-DCFPyL PET/CT and outcome data available. Inclusion and exclusion criteria for the EAP are provided in the supplemental materials (available at http://jnm.snmjournals.org). In brief, eligible patients with mCRPC had PSMA-positive lesions defined by VISION criteria and had previously received at least 1 androgen receptor signaling inhibitor and 1 taxane-based chemotherapy regimen (1). In addition, eligible patients must have shown adequate organ function and could not receive concurrent cytotoxic chemotherapy, immunotherapy, radiopharmaceutical therapy, or investigational therapy. Eventually, patients enrolled in the EAP were administered 7.4 GBq (200 mCi) ± 10% of ^177^Lu-PSMA every 6 wk for up to 6 cycles. Treatment cycles continued until either disease progression, severe toxicity, or patient withdrawal. This cohort partially overlaps with a previously published EAP study that focused on efficacy and toxicity (3), whereas the present analysis aimed to evaluate imaging biomarkers. The institutional review boards approved this retrospective imaging analysis and waived the study-specific consent requirement (University of California Los Angeles #21-001565). As 27 patients had undergone PSMA PET/CT before referral to the 3 participating institutions, their pretreatment scans were obtained at various imaging facilities. Therefore, the reconstruction parameters varied because of differences in scanner models and imaging protocols. We collected the predefined clinical data from patient medical records at each participating institution.

### Image Analysis

#### Visual Indicator Analysis

One reader who is dual board–certified in radiology and nuclear medicine, masked to the patient outcomes, reviewed the baseline PSMA PET/CT scan of each patient to apply the visual PSMA PET tumor–to–salivary gland ratio (vPSG) and heterogeneity and intensity of tumors (HIT) score (11,12). vPSG scores are criteria using the visual uptake of parotid glands as an organ of reference and are categorized into 3 groups (high, most of the lesions showed higher uptake than the parotid glands; intermediate, neither low nor high; low, most of the lesions showed lower uptake than the parotid glands). HIT scores are derived from a combination of heterogeneity of the whole-body uptake and SUV_max_ of the most intense lesions and are categorized into 4 groups (SUV_max_, <15; SUV_max_, 15–79 with heterogeneous intensity; SUV_max_, 15–79 with homogeneous intensity; SUV_max_, ≥80). In the case of a controversial assessment for visual analysis, a second reader, with over 8 y of experience in reading PSMA PET/CT, assessed the score.

#### Quantitative Indicator Analysis

Quantitative analysis was performed for whole-body tumor burden and parotid glands on PSMA PET/CT using a semiautomatically contouring Affinity system (Hermes Medical Solutions) by 3 experienced nuclear medicine physicians. For the segmentation of lesions, a specific threshold, which was described elsewhere, derived using a 3-cm spheric volume of interest on normal liver parenchyma, was used: threshold of SUV = (4.3 ÷ liver SUV_mean_) × (liver SUV_mean_ + SD) (13). After the first autosegmentation was conducted for areas with the SUV greater than the used threshold value, nontumorous uptakes were manually excluded. Quantitative parameters for the parotid glands were obtained from the volume of interest of parotid glands during the tumor segmentation process. We derived the following quantitative metrics, which might be prognostic parameters for patients with ^177^Lu-PSMA, from segmentation data of total tumor burden: total tumor volume (TTV), total tumor SUV_mean_, total tumor SUV_max_, total lesion uptake (TLU) (TTV × SUV_mean_), total lesion quotient (TLQ = TTV ÷ SUV_mean_), and quantitative PSG (qPSG) (7,11).

### Outcome Data

The outcome data included a 50% or greater PSA decline (PSA50) relative to baseline at any time during ^177^Lu-PSMA treatment (best response), PSA PFS, and OS. PSA PFS was the period from treatment initiation to PSA progression or death from any cause, whichever occurred first, based on the Prostate Cancer Clinical Trials Working Group 3 criteria (14). OS was calculated from 4 wk before treatment initiation to death of any cause or the last follow-up alive. The rationale for starting the time of OS at 4 wk before treatment initiation was to follow the OS calculation in the VISION trial, that is, from the time of randomization. The outcome data were collected in November 2024.

### Statistical Analysis

Baseline patient characteristics, clinical parameters, and imaging-based indicators were summarized using median and interquartile range (IQR) for continuous variables and counts with percentages for categoric variables. To compare imaging indicators between PSA50 responders and nonresponders, the Wilcoxon rank-sum test was used for continuous variables and the Fisher exact test was used for categoric variables. To visualize and evaluate associations between imaging biomarkers and outcomes, Kaplan–Meier survival analyses were performed for PFS and OS, with comparisons assessed using the log-rank test. Quantitative imaging indicators were stratified into tertiles (low, intermediate, high), and visual scores (vPSG and HIT) were categorized based on established scoring systems (11,12). For qPSG, cutoffs were defined a priori from the original publication (11): low (≤0.5), intermediate (>0.5 to < 1.5), and high (≥1.5). Univariable Cox proportional hazards (PH) models were used to assess the association between each imaging indicator and both PSA PFS and OS, with concordance index (c-index) values reported to evaluate model discrimination. Multivariable Cox PH models were subsequently fit to determine whether imaging indicators added prognostic value beyond clinical variables. These models were adjusted for age, time since prostate cancer diagnosis, number of prior systemic therapies, baseline hemoglobin, thrombocyte count, and PSA. *P* values are reported for the imaging term, and hazard ratios (HRs) for continuous variables are scaled by the IQR (Q1–Q3) for interpretability. Receiver operating characteristic curve analyses were performed to assess the ability of each imaging indicator to predict PSA50 response, with the area under the curve and 95% CIs used to evaluate discriminative performance. For exploratory purposes, optimal cut-points for imaging biomarkers were derived using conditional inference survival tree analysis. This method partitions the data based on statistically significant differences in PFS and OS and is useful for detecting nonlinear or threshold effects. Median survival times were computed for each tree-defined subgroup. Given the potential for overfitting, especially in smaller samples, these tree-based findings should be interpreted cautiously and validated in independent cohorts. All statistical analyses were performed using R version 4.0.3 (R Foundation for Statistical Computing; https://www.r-project.org/). The 2-tailed *P* values of less than 0.05 were considered statistically significant.

## RESULTS

### Patient Characteristics

**FIGURE 1.**
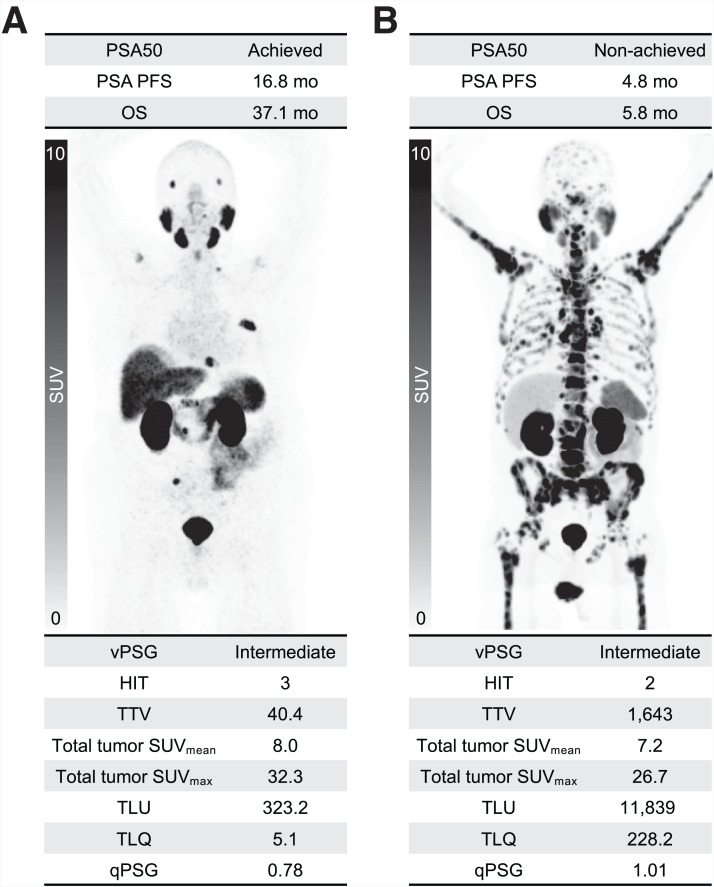
Baseline PSMA PET of 2 patients with PSA50 achieved and long survival (A) and PSA50 nonachieved and short survival (B) periods after ^177^Lu-PSMA therapy. Maximum-intensity projections are displayed with SUV range of 0–10. Baseline values of the quantitative and visual indicators are provided.

In total, 88 patients with mCRPC who received ^177^Lu-PSMA with available pretherapeutic PSMA PET/CT within the EAP at participating institutions between May 2021 and March 2022 were included in this analysis. Sixty-four (73%) and 24 (27%) patients were scanned with [^68^Ga]Ga-PSMA-11 and [^18^F]F-DCFPyL PET/CT, respectively. The median time between baseline PSMA PET/CT and the first cycle of ^177^Lu-PSMA was 7.6 wk (IQR, 5.9–12.0 wk). The patients’ characteristics, summary of imaging indicators, and clinical outcomes are shown in Table 1. All patients received a median of 4 doses (IQR, 2–6 doses) with median cumulative injected activity of 24.8 GBq (IQR, 14.5–43.7 GBq) of ^177^Lu-PSMA. The median follow-up time was 36.1 mo (95% CI, 33.8–37.8 mo). The PSA50 achievement rate was 43%, and the number of PSA PFS events was 86 with a median PSA PFS of 4.5 mo (95% CI, 3.7–7.2) and a 2-y PSA PFS rate of 7% (95% CI, 3–16%). The number of deaths was 67 with a median OS of 12.5 mo (95% CI, 10.4–17.1 mo) and a 2-y OS rate of 29% (95% CI, 20–40%). Figure 1 shows each value of the quantitative and visual indicators for the representative baseline PSMA PET in 2 patients with different survival periods.

**TABLE 1.** Patient Characteristics, Imaging Indicators, and Clinical Outcome

Characteristic	Value
*n*	88
Age (y)	71 (65–78)
Time since diagnosis (y)	8.7 (4.8–14.6)
No. of prior systemic therapy	4 (3–5)
Hemoglobin (g/dL)*	11.3 (9.5–12.2)
Thrombocytes (×10^9^/L)^†^	220 (157–297)
PSA (ng/mL)	96.5 (17.1–323.7)
Visual indicators	
vPSG score	
Low	15 (17%)
Intermediate	28 (32%)
High	45 (51%)
HIT score	
1	16 (18)
2	27 (31)
3	26 (30)
4	19 (21)
Quantitative indicators	
TTV (mL)	283.5 (85.9–654)
Total tumor SUV_mean_	9.1 (6.6–12.0)
Total tumor SUV_max_	38.4 (24.5–76.2)
TLU	2,478.4 (823.5–7,129.6)
TLQ	27.8 (8.4–76.8)
qPSG	1.17 (0.80–1.65)
Cycles of ^177^Lu-PSMA received	4 (2–6)
Follow-up (mo)	36.1 (33.8–37.8)
No. of PSA decline ≥ 50%^‡^	38 (43)
No. of PSA PFS events	86 (98)
PSA PFS (mo)	4.5 (3.7–7.2)
2-y PSA PFS rate (%)	7 (3–16)
No. of OS events	67 (76)
OS (mo)	12.5 (10.4–17.1)
2-y OS rate (%)	29 (20–40)

* Data are missing for 5 patients.

† Data are missing for 6 patients.

‡ Five patients who are missing posttreatment PSA data and 1 patient whose baseline PSA was <0.01 were counted as negative for PSA decline ≥ 50%.

Qualitative data are number with percentage; continuous data are median with interquartile range or, for follow-up time, PSA PFS, and OS data, median with 95% CI.

### PSA50 and Imaging Indicators

Patients who achieved a PSA50 response exhibited a significantly higher prevalence of high vPSG scores and lower frequencies of intermediate and low scores compared with nonresponders (high, 74% vs. 34%; intermediate, 21% vs. 40%; low, 5% vs. 26%; *P* < 0.001 for overall distribution). The PSA50 responder group demonstrated a significantly lower frequency of low HIT scores and a higher frequency of elevated scores compared with nonresponders (score 1, 5% vs. 28%; score 2, 24% vs. 36%; score 3, 39% vs. 22%; score 4, 32% vs. 14%; *P* = 0.005 for overall distribution).

The PSA50 achievement group showed values of total tumor SUV_mean_ and total tumor SUV_max_ significantly higher than those of the PSA50 nonachievement group (11.5 vs. 7.0 and 56.5 vs. 27.3, respectively; *P* < 0.001 for both) and a value of qPSG that was significantly lower than that of the PSA50 nonachievement group (1.45 vs. 0.99; *P* < 0.001). No significant difference in TTV, TLU, and TLQ was observed between PSA50 achievement and nonachievement groups.

The waterfall plots display the relationship between imaging indicators and PSA changes in patients with complete PSA data (Supplemental Figs. 1 and 2). For PSA50’s predictive performance, the total tumor SUV_mean_ achieved the highest area under the curve of 0.81 (95% CI, 0.73–0.90). Supplemental Table 1 provides details of the association between PSA50 and imaging indicators.

### PSA PFS and Imaging Indicators

**FIGURE 2.**
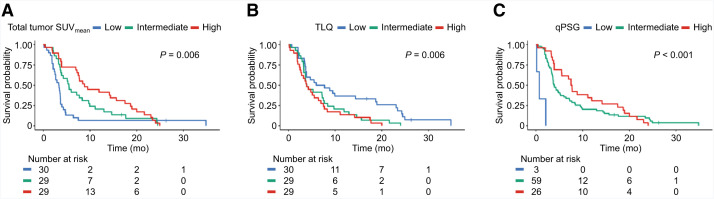
Kaplan–Meier curves (log-rank tests) of PSA PFS for each imaging indicator: (A) total tumor SUV_mean_, (B) TLQ, and (C) qPSG.

vPSG and HIT scores showed a visual trend of longer PSA PFS with increasing score categories (i.e., low < intermediate < high for vPSG and 1 < 2 < 3 < 4 for HIT); however, this trend was not statistically significant on the log-rank test (Supplemental Fig. 3). The total tumor SUV_mean_, TLQ, and qPSG were statistically significantly associated with PSA PFS (log-rank test, *P* = 0.006, *P* = 0.006, and *P* < 0.001, respectively) (Fig. 2). The corresponding HR and c-index are shown in Table 2. For univariate analysis, the total tumor SUV_mean_ was a statistically significant positive prognosticator and achieved the highest c-index of 0.678 (HR, 0.56; 95% CI, 0.38–0.83; *P* = 0.004), followed by the total tumor SUV_max_ as a positive prognosticator with a c-index of 0.640 (HR, 0.69; 95% CI, 0.49–0.97; *P* = 0.034). The other univariate Cox PH regression models were not statistically associated with the PSA PFS.

**TABLE 2.** Univariate and Multivariate Cox PH Regression Analysis for PSA PFS

	Univariate			Multivariate*		
Indicator	HR	*P*	c-Index	HR	*P*	c-Index
vPSG score		0.066	0.600		0.016	0.640
Low	Reference			Reference		
Intermediate	1.25 (0.63–2.48)	0.520		1.35 (0.67–2.72)	0.404	
High	0.70 (0.37–1.33)	0.279		0.62 (0.32–1.20)	0.156	
HIT score		0.100	0.596		0.269	0.639
1	Reference			Reference		
2	1.18 (0.60–2.32)	0.634		1.03 (0.50–2.11)	0.938	
3	0.66 (0.33–1.31)	0.236		0.67 (0.33–1.33)	0.249	
4	0.62 (0.31–1.27)	0.192		0.60 (0.29–1.24)	0.167	
TTV	1.08 (0.91–1.27)	0.397	0.511	1.01 (0.83–1.23)	0.907	0.597
Total tumor SUV_mean_	0.56 (0.38–0.83)	0.004	0.678	0.58 (0.39–0.86)	0.007	0.667
Total tumor SUV_max_	0.69 (0.49–0.97)	0.034	0.640	0.71 (0.50–1.01)	0.056	0.637
TLU	1.02 (0.86–1.21)	0.816	0.475	0.98 (0.81–1.18)	0.822	0.598
TLQ	1.15 (0.97–1.35)	0.107	0.556	1.08 (0.88–1.32)	0.473	0.597
qPSG	0.76 (0.54–1.07)	0.118	0.628	0.61 (0.42–0.89)	0.010	0.656

* Adjusted by 6 clinical variables as follows: patient age, time since initial diagnosis of prostate cancer, number of prior systemic therapy, count of hemoglobin, count of thrombocytes, and baseline PSA. c-Index of Cox PH regression model, which only incorporated the 6 clinical variables, was 0.594.

For continuous variables, HRs are expressed per interquartile range, from Q1–Q3, with 95% CIs in parentheses.

For multivariate analysis adjusted for clinical variables, the total tumor SUV_mean_ was identified as a positive prognosticator with the highest c-index of 0.667 (HR, 0.58; 95% CI, 0.39–0.86; *P* = 0.007), followed by the qPSG as a positive prognosticator with a c-index of 0.656 (HR, 0.61; 95% CI, 0.42–0.89; *P* = 0.010). The vPSG score showed the third highest c-index of 0.640; however, when using score “low” as the reference, the HR did not exhibit a sequential trend across ordinal categories as anticipated. The c-index of multivariate Cox PH regression models of these top 2 significant quantitative indicators for PSA PFS outperformed the c-index of the model incorporating clinical variables alone (0.667, 0.656 vs. 0.594). In accordance with the result, the univariate confidential inference survival tree, as an exploratory initiative, was adapted to the total tumor SUV_mean_, and a cutoff value of 7.8 was derived (≤7.8 for 3.3 mo vs. >7.8 for 7.7 mo for PSA PFS; *P* = 0.004) (Supplemental Fig. 4).

### OS and Imaging Indicators

**FIGURE 3.**
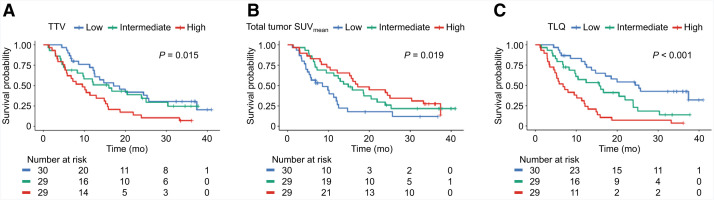
Kaplan–Meier curves (log-rank tests) of OS for each imaging indicator: (A) TTV, (B) total tumor SUV_mean_, and (C) TLQ.

HIT scores showed a significant *P* value on the log-rank test; however, the median OS of each score did not exhibit a sequential trend across ordinal categories as anticipated (Supplemental Fig. 5). The TTV, total tumor SUV_mean_, and TLQ were statistically significantly associated with OS (log-rank test, *P* = 0.015, *P* = 0.019, and *P* < 0.001, respectively) (Fig. 3). The corresponding HR and c-index are shown in Table 3. For univariate analysis, the TLQ was a statistically significant negative prognosticator and achieved the highest c-index of 0.658 (HR, 1.38; 95% CI, 1.17–1.63; *P* < 0.001), followed by the HIT score with a c-index of 0.639; however, when using score “1” as the reference, the HR did not exhibit a sequential trend across ordinal categories as anticipated. The total tumor SUV_mean_ and TTV were statistically significant positive and negative indicators and showed the third and fourth highest c-index of 0.634 (HR, 0.52; 95% CI, 0.33–0.81; *P* = 0.004) and 0.621 (HR, 1.28; 95% CI, 1.08–1.52; *P* = 0.005). The other univariate Cox HR regression models were not statistically associated with OS.

**TABLE 3.** Univariate and Multivariate Cox PH Regression Analysis for OS

	Univariate			Multivariate*		
Indicator	HR	*P*	c-Index	HR	*P*	c-Index
vPSG score		0.300	0.589		0.085	0.685
Low	Reference			Reference		
Intermediate	1.23 (0.57–2.61)	0.598		1.53 (0.69–3.38)	0.298	
High	0.79 (0.39–1.59)	0.504		0.78 (0.38–1.61)	0.497	
HIT score		0.010	0.639		0.078	0.688
1	Reference			Reference		
2	1.67 (0.80–3.51)	0.174		1.54 (0.69–3.43)	0.290	
3	0.58 (0.27–1.26)	0.168		0.61 (0.27–1.39)	0.241	
4	0.87 (0.39–1.95)	0.740		0.92 (0.40–2.09)	0.838	
TTV	1.28 (1.08–1.52)	0.005	0.621	1.19 (0.98–1.45)	0.081	0.667
Total tumor SUV_mean_	0.52 (0.33–0.81)	0.004	0.634	0.54 (0.34–0.86)	0.009	0.687
Total tumor SUV_max_	0.83 (0.57–1.22)	0.340	0.558	0.89 (0.60–1.32)	0.561	0.669
TLU	1.19 (0.99–1.42)	0.055	0.584	1.13 (0.93–1.36)	0.218	0.662
TLQ	1.38 (1.17–1.63)	<0.001	0.658	1.30 (1.05–1.60)	0.014	0.682
qPSG	0.91 (0.63–1.31)	0.597	0.545	0.68 (0.46–1.00)	0.051	0.672

* Adjusted by 6 clinical variables as follows: patient age, time since initial diagnosis of prostate cancer, number of prior systemic therapy, count of hemoglobin, count of thrombocytes, and baseline PSA. c-Index of Cox PH regression model, which only incorporated the 6 clinical variables, was 0.661.

For continuous variables, HR are expressed per interquartile range, from Q1 to Q3, with 95% CIs in parentheses.

For multivariate analysis adjusted for clinical variables, the total tumor SUV_mean_ was identified as a positive prognosticator with the highest c-index of 0.687 (HR, 0.54; 95% CI, 0.34–0.86; *P* = 0.009), followed by the TLQ as negative prognosticator with a c-index of 0.682 (HR, 1.30; 95% CI, 1.05–1.60; *P* = 0.014). The c-index of multivariate Cox PH regression models of these top 2 significant quantitative indicators for OS outperformed the c-index of the model incorporating clinical variables alone (0.687, 0.682 vs. 0.661). In accordance with the result, the univariate confidential inference survival tree, as an exploratory initiative, was adapted to the total tumor SUV_mean_, and a cutoff value of 7.8 was derived (≤7.8 for 6.8 mo vs. >7.8 for 16.6 mo for OS; *P* = 0.003) (Supplemental Fig. 6).

## DISCUSSION

In our analysis within the EAP cohort, the total tumor SUV_mean_ demonstrated the highest value to discriminate PSA50 achievement after initiation of ^177^Lu-PSMA therapy in patients with mCRPC, outperforming other imaging biomarkers. Furthermore, total tumor SUV_mean_ was identified as the most robust prognosticator for both PSA PFS and OS, even after adjusting for clinical variables by multivariate analysis. These findings suggest that favorable profiles for ^177^Lu-PSMA therapy are not always captured by basic clinical information, such as age and serum PSA level, among other factors, and the total tumor SUV_mean_ derived from baseline PSMA PET/CT can add meaningful prognostic value beyond clinical information alone.

Various quantitative indicators can be derived from baseline PSMA PET/CT; however, only a limited number of these parameters have demonstrated prognostic relevance. Total tumor SUV_max_ showed a difference between the PSA50 achievement group and the nonachievement group, but no association with OS was observed. This finding regarding OS was consistent with a report of secondary analysis of the VISION trial and supported the notion that using only the most avid lesion for treatment selection is insufficient in the context of PSMA radiopharmaceutical therapy (15). Although TTV did not correlate with PSA response, it showed a significant association with OS. This finding is not limited to ^177^Lu-PSMA therapy for mCRPC but is also in line with the general oncologic understanding that a high tumor burden on PET imaging is associated with poor prognosis (7,16–18). Given that greater TTV is associated with shorter OS, whereas higher SUV_mean_ correlates with improved OS, it is conceptually reasonable to assess prognosis using a composite imaging biomarker that integrates both tumor burden and tracer uptake. The TLQ, which incorporates both volumetric and intensity-based information, exemplifies such an integrative metric. In line with a previous study, TLQ demonstrated the greatest prognostic reliability for OS in univariate analysis (7,19,20). However, contrary to expectations, total tumor SUV_mean_ demonstrated greater prognostic reliability than TLQ in the multivariate analysis that incorporated clinical variables. These findings suggest that, in real-world clinical practice, total tumor SUV_mean_ may serve as a more practical and reliable imaging biomarker than TLQ, as it appears less susceptible to multicollinearity and confounding by other clinical variables.

Visual assessment indicators such as vPSG and HIT scores did not demonstrate a strong association with outcome measures after ^177^Lu-PSMA therapy in patients with mCRPC, and we were unable to reproduce the findings reported in previous studies (11,12). Factors such as cohort size and reader experience may have had an influence on the reproducibility of visual assessments, reflecting the subjective nature of visual evaluation. However, even though the performance of visual indicators in our analysis did not meet expectations, there is no doubt that visual assessment remains clinically valuable. The VISION trial used visual assessment for screening patients effectively, and the VISION PET criteria have demonstrated their utility in predicting response to ^177^Lu-PSMA therapy. The VISION PET screening–failed patients had worse outcomes than did the VISION PET–eligible patients (21). Therefore, whereas visual indicators are useful, involving multiple reviewers is crucial to enhance consistency and accuracy, especially when recommending against ^177^Lu-PSMA therapy based on the visual assessment, such as vPSG or HIT scores.

The exploratory cutoff value of 7.8 for total tumor SUV_mean_ appeared to be prognostic for both PSA PFS and OS in our cohort. The secondary analysis of the VISION trial showed that participants receiving ^177^Lu-PSMA with an SUV_mean_ of 8 or greater had longer OS than those with an SUV_mean_ of less than 8 (HR, 0.65; 95% CI, 0.52–0.81; *P* < 0.001), with a median OS of 13.6 mo in the group with an SUV_mean_ of less than 8 (5). In our analysis, patients with a total tumor SUV_mean_ of 7.8 or less had a median OS of only 6.8 mo, which was markedly shorter than that reported in the VISION trial at this similar cutoff point. This discrepancy, despite adherence to the same VISION PET eligibility criteria, may be attributed to differences in the purpose of patient recruitment between a clinical trial and an EAP. The EAP cohort may have included a greater proportion of patients with late-stage mCRPC, who were more likely to exhibit lower total tumor SUV_mean_ values. Supporting this interpretation, Thang et al. reported in their small observational study (*n* = 16) that patients who were ineligible for ^177^Lu-PSMA because of low PSMA expression or discordant [^18^F]FDG-positive PSMA-negative disease had a low SUV_mean_ of 5.6 and a median OS of only 2.5 mo (22). In the TheraP trial, the imaging screen–failed patient (if lack of metastatic lesions with SUV_max_ ≥ 20, or if any measurable lesions with SUV_max_ ≤ 10, or if discordant lesions with FDG-positive PSMA-negative) showed a poorer outcome and lower SUV_mean_ values than the eligible patient (restricted mean survival time for OS: 11.0 vs. 18.8 mo, *P* < 0.0001; median SUV_mean_, 5.3 vs. 8.5, *P* < 0.001) (6). Collectively, these findings suggest that patients with advanced-stage disease and low PSMA expression may derive only limited OS benefit from ^177^Lu-PSMA.

The total tumor SUV_mean_ may be prognostically informative when integrated into clinical practice; however, its calculation requires dedicated software and is therefore not yet widely available. In practical settings, visual assessments on maximum-intensity-projection images—such as the vPSG or HIT score—remain useful alternatives for estimating overall PSMA expression. Accordingly, these measures can be used selectively depending on the resources and workflow available at each institution. Importantly, these measures should support risk stratification and individualized treatment planning rather than serve as strict eligibility criteria for ^177^Lu-PSMA therapy, as therapeutic options for mCRPC remain limited. For patients with advanced disease and low total tumor SUV_mean_ value, or visually low PSMA uptake, treatment decisions should consider the patient’s overall clinical status and tumor biology. Options may include dose-intensified or combination ^177^Lu-PSMA regimens, PARP inhibitors in BRCA-mutated disease, or α-emitting radiopharmaceutical therapy. The ongoing phase 2 prospective LPS-Boost trial (NCT06526299) evaluating double-dose ^177^Lu-PSMA in patients with a total tumor SUV_mean_ of less than 10 is expected to inform management strategies for patients with lower PSMA-uptake disease (23).

This study has several limitations. First, the sample size was relatively small, and PET scanners and reconstruction methods were not harmonized, which may have limited the statistical power and the reproducibility of the findings. In fact, digital PET/CT systems and deep learning–based image reconstruction—both increasingly adopted in recent years—are known to yield higher SUV metrics than analog PET/CT or conventional model-based iterative reconstruction (24,25). Consequently, it is uncertain whether external cohorts that include PET images acquired with such advanced technologies would be able to reproduce our results. On the other hand, the scanner heterogeneity within our cohort reflects real-world clinical practice, where imaging is performed using diverse equipment across centers. Including multiple scanner types may enhance generalizability by demonstrating how total tumor SUV_mean_ behaves under routine clinical conditions. Additionally, results from prior phantom-based methodologic studies suggest that scanner-related variability in SUV metrics does not necessarily translate into clinically meaningful differences—particularly for SUV_mean_, which is generally more stable than SUV_max_ (26,27). Nevertheless, scanner heterogeneity may have introduced additional noise into our measurements. Future studies with harmonized imaging protocols or cross-calibration across centers, ideally in a larger cohort, will be important to confirm the robustness of total tumor SUV_mean_. Second, we did not assess inter- and intraobserver variability in the quantitative indicator analysis, which may have influenced the consistency and reproducibility of the results. Third, visual assessments were conducted by a single reader, albeit with review by a senior nuclear medicine physician. Although this approach ensured consistency, involving multiple independent readers would enhance the reliability of visual interpretation. Fourth, the multivariable Cox PH model included only 6 clinical variables. This limited scope may not fully capture the complexity of real-world clinical decision-making or patient heterogeneity. Finally, although we proposed an exploratory cutoff value for total tumor SUV_mean_ from survival tree analysis, it was used only to support statistical modeling and illustrate potential prognostic trends, not for clinical decision-making at this stage. As noted in the first limitation, because SUV_mean_ varies across scanners, protocols, and patient populations referred for ^177^Lu-PSMA therapy, optimal cutoffs will differ by institution. Without external validation, this cutoff should be regarded as hypothesis-generating, and additional studies are needed before any cutoff could be considered for clinical use.

## CONCLUSION

In our analysis within the EAP cohort, quantitative indicators had superior reliability compared with visual indicators in predicting the outcome of mCRPC with ^177^Lu-PSMA therapy. In particular, total tumor SUV_mean_ was identified as the most robust prognostic indicator for both PSA PFS and OS after ^177^Lu-PSMA therapy, and it offers value in addition to basic patient information for clinical assessment. Incorporating the imaging indicator into clinical decision-making for pre–^177^Lu-PSMA therapy could aid in patient selection and treatment planning.

## DISCLOSURE

Koichiro Kimura received the 2024 SNMMI Wagner–Torizuka Fellowship. Adrien Holzgreve is funded by the Deutsche Forschungsgemeinschaft (DFG, German Research Foundation), 545058105. He also reports compensation for scientific consulting by ABX Advanced Biochemical Compounds outside the submitted work. Lilja Solnes receives research funding from Novartis, Perspective Therapeutics, Cellectar, Curium, and the U.S. Department of Defense and receives royalties from Elsevier. Andrei Gafita was supported by the Prostate Cancer Foundation (21YOUN18). Jeremie Calais reports grants from support to his institution from Lantheus, Novartis, and POINT Biopharma. He also reports consulting activities (advisory boards, speaker, blinded reader) for Advanced Accelerator Applications, Amgen, Astellas, Bayer, Blue Earth Diagnostics Inc., Curium Pharma, Coretag, DS Pharma, Fibrogen, GE HealthCare, Isoray, IBA RadioPharma, Janssen Pharmaceuticals, Monrol, Lightpoint Medical, Lantheus, Novartis, Nucleus Radiopharma, Pfizer, POINT Biopharma, Progenics, Radiomedix, Radiopharm Theranostics, Sanofi, Siemens-Varian, SOFIE, and Telix Pharmaceuticals, outside of the submitted work. Johannes Czernin is a founder of SOFIE Biosciences and holds equity in the company and in intellectual property invented by him, patented by the University of California, and licensed to SOFIE Biosciences. He is a founder and board member of Trethera Therapeutics and holds equity in the company and in intellectual property invented by him, patented by the University of California, and licensed to Triangle. He serves on the medical advisory board of Actinium Pharmaceuticals and on the scientific advisory boards of POINT Biopharma, RayzeBio, and Aktis Oncology. No other potential conflict of interest relevant to this article was reported.

## Notes

KEY POINTS **QUESTION:** Which baseline PSMA PET/CT–derived imaging biomarkers most reliably predict outcomes and provide added prognostic value beyond baseline clinical parameters prior to ^177^Lu-PSMA therapy? **PERTINENT FINDINGS:** Among the evaluated imaging metrics, the total tumor SUV_mean_ demonstrated the strongest predictive performance for achieving PSA50 and remained a robust independent prognosticator for both PSA PFS and OS after adjustment for clinical variables by multivariate analysis. It outperformed other visual and quantitative PET imaging–derived biomarkers. **IMPLICATIONS FOR PATIENT CARE:** The total tumor SUV_mean_ derived from baseline PSMA PET/CT had prognostic value for outcomes of ^177^Lu-PSMA. It may guide patient selection decisions in conjunction with clinical information.
